# Green synthesized AgNPs as a probe for colorimetric detection of Hg (II) ions in aqueous medium and fluorescent imaging in liver cell lines and its antibacterial activity

**DOI:** 10.1186/s11671-024-04014-8

**Published:** 2024-05-02

**Authors:** Sanjana Tewari, Shalini Sahani, Neetu Yaduvanshi, Ritu Painuli, Nalini Sankararamakrishnan, Jaya Dwivedi, Swapnil Sharma, Sung Soo Han

**Affiliations:** 1 Department of Chemistry, Banasthali Vidyapith, Banasthali, Rajasthan 304 022 India; 2 School of Chemical Engineering, Yeungnam University, 280 Daehak-ro, Gyeongsan, 38541 South Korea; 3 Department of Chemistry, School of Applied and Life Sciences, Uttaranchal University, Dehradun, Uttarakhand 248007 India; 4 Centre for Environmental Science and Engineering, Indian Institute of Technology, Kanpur, Uttar Pradesh 208016 India; 5 Department of Pharmacy, Banasthali Vidyapith, Banasthali, Rajasthan 304022 India

**Keywords:** Persimmon leaves extract, Silver nanoparticles, Mercuric ion, Colorimetric detection, Fluorescence imaging, Antibacterial activity

## Abstract

The present research aimed at green synthesis of Ag nanoparticles (AgNPs) based colorimetric sensor using persimmon leaf extract (PLE) for selective detection of mercuric ion (Hg^2+^). Optimization of reaction conditions viz. pH, concentration of PLE, time was done and further AgNPs were characterized using UV, IR, FE-SEM, EDX, XRD and TEM analysis. The developed AgNPs were evaluated for the selective colorimetric detection of Hg^2+^ in aqueous medium and fluorescence imaging of Hg^2+^ ions in liver cell lines. Later, the antibacterial activity of AgNPs was performed against *S. aureus* and *E. coli.* The findings of the study revealed that PLE mediated AgNPs exhibited notable limit of detection up to 0.1 ppb, high efficiency, and stability. The antibacterial study indicated that developed AgNPs has impressive bacterial inhibiting properties against the tested bacterial strains*.* In conclusion, developed biogenic AgNPs has high selectivity and notable sensitivity towards Hg^2+^ ions and may be used as key tool water remediation.

**Graphical abstract:**

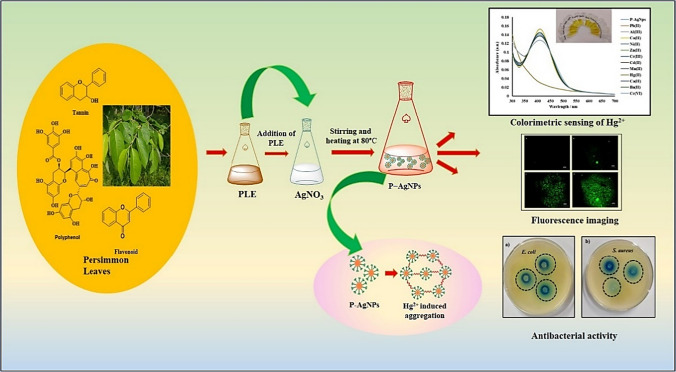

**Supplementary Information:**

The online version contains supplementary material available at 10.1186/s11671-024-04014-8.

## Introduction

Pollution in water bodies by various agents including heavy metals and microbial species poses a serious threat to society. The pollution of water resources mainly due to heavy metal ions is a top global concern. Most of the heavy metal ions are directly released from industrialized areas, thus worsening the quality of water [16]. Among all the heavy metals, mercury (Hg^2+^) ions are one such persistent contaminant that is associated with bioaccumulation in aquatic life that severely affects the environment. Even low exposure to mercury may lead to harmful effects on human health such as neuronal, hepatic, memory loss, nephritic damage, and decreased fertility rate, as well as defects in offspring birth in living organisms. The main source of mercury pollution is the burning of coal in power production [12] as well as natural sources such as volcanic eruptions contribute to the elevated levels of mercury in ambient water, air, and even food [26]. Owing to these issues, the development of a green and cost-effective method for sensing Hg^2+^ received significant attention from investigators worldwide [29] to regulate the permissible minimum concentration of Hg^2+^ in drinking water which is about 2 µg L^−1^ [17].

Various analytical techniques viz. like atomic absorption or fluorescence spectrometry [7, 24], inductive coupled plasma mass spectroscopy [34], chromatography coupled mass spectroscopy, and electro-analytical techniques [5], are commonly employed in the sensing or quantification of Hg^2+^ in the environment [6]. However, these methods are frequently associated with several issues and requirements namely complex sample treatment, storage, well-trained operators, usage of toxic chemicals and complicated synthesis processes making it difficult for on-site application [25, 28, 51]. As a result, the development of a sensitive, selective, effective, and label-free detection method for Hg^2+^ ions is critical at this juncture [19]. Recently, colorimetric sensors have been found to exhibit promising detection abilities towards several materials and pollutants and gaining considerable attention from investigators due to their quick detection, easy fabrication, cost-effectiveness, high selectivity, and visual sensing [32].

Numerous experimental techniques have been documented for Hg^2+^ detection utilized nanozyme [41], fluorophores [53], thiol-containing ligand [10, 11], tyrosine [6, 7], paper electrodes [10, 11]. Although these approaches are very sensitive and selective, they have a number of disadvantages, such as the poor aqueous solubility of fluorophores, the use of hazardous chemicals, non-polar solvents, and artificial additives or capping agents, which limits their applicability.

Along with the toxic metal ions, microbial species viz. *E. coli and S. aureus* act as significant pollutants for contamination of water resources that cause deleterious effects on humankind. Conventional disinfection methods like ozonation and chlorination have been used extensively for water disinfection however, these methods have their limitations. The presence of halogenated disinfection byproducts (DBPs) in chlorinated waters impart significant toxicity on chronic exposure and causes deleterious effects in the recipients. Many DBPs cause cancer in humans, and some are mutagenic genotoxic, or cytotoxic. Furthermore, Ozone reacts with water matrix components, resulting in the formation of oxidation byproducts, some of which can cause adverse health effects [33]. Thus, to circumvent these limitations, the development of alternative approaches is of paramount importance in current times [36, 37, 42]. In regards to this, the present study is an attempt to evaluate the developed sensor for its disinfectant activity in the aqueous systems. The gold and silver nanocomposite have been reported for sensing of Hg^2+^ with a lower limit of detection (LOD) owing to a high extinction coefficient. When compared to other metal nanoparticles of the same size, highly inexpensive silver nanoparticles have a substantial extinction coefficient [44, 50]. AgNPs lend themselves well to redox and aggregation-based chemistry for the sensitive and selective detection of hazardous metal ions. In this way, high sensing characteristics were observed by AgNPs towards targeted metal ions [21]. The plasmonic band of AgNPs relies on the size and the distance between the ions, which makes them superior in eye-distinguishable visual sensors [1, 22, 38].

The clean, green, and sustainable approaches for the preparation of silver nanoparticles using microorganisms (algae, fungi, etc.) and plant extracts provide key attributes over existing physical and chemical methods [2, 45]. The extract of plants contains various metabolites such as aldehyde, ketone, protein, polysaccharides, terpenoids, flavonoids, tannins, alkaloids, flavonoids, and amines help in the stabilization and reduction of NPs via capping [15, 27, 35].

Persimmon (*Diospyros kaki* L.) belongs to the family Ebenaceae and has been acknowledged for impressive medicinal properties viz. treatment of angina, ischemia stroke, internal haemorrhage, hypertension, and some infectious diseases, etc. [31]. Persimmon leaves are rich in bioactive components such as proanthocyanidins, flavonoid, tannins, phenols, catechin, etc. [52]. Moreover, compared with other kinds of fallen leaves, persimmon-fallen leaves contain the higher amount of tannins. The functional groups of persimmon derived tannins have considerable affinity for heavy metal ions. Besides, polyphenols are abundant in persimmon that contains multiple phenolic hydroxyl groups linked to one or more benzene ring systems which in turn allows impressive sensitivity towards metal ions [14].

Considering these facts, the present study has aimed to synthesize AgNPs using persimmon leaf extract (PLE) and to evaluate them as effective visual sensors for toxic metal ion Hg^2+^. Notably, the developed sensor is easy to implement, environmentally friendly, and does not require any sophisticated instrument. The developed sensor demonstrated improved sensitivity of up to 0.1 ppb and effective sensing of Hg^2+^ in tap water with simultaneous fluorescent cell imaging in liver tissue cells and inhibition of pathogenic bacterial species, implying broad application prospects for tracking Hg^2+^ levels in natural environments.

## Experimental

### Chemical and materials

Fresh persimmon leaves were collected from Nainital, Uttarakhand, India. Silver nitrate (AgNO_3_) and heavy metal salts were purchased from Sigma-Aldrich (India) and used unpurified. The aqua-regia and Milli-Q water were used to wash and rinsed the glassware before use. Milli-Q and deionized water were used throughout the experimental procedure.

### Preparation of persimmon leaf extract

Persimmon leaves were properly washed and cleaned with Milli-Q water and chopped the leaves into small pieces. The small pieces of leaves were dried in an oven at 50 °C. An aqueous extract of persimmon leaves was made by combining 3 g of dried fine powder with 100 mL Milli-Q water and heating the mixture at approximately 40℃ for 4 h when the solution of color changed to brown. The formed PLE was cooled at room temperature, filtered, and stored in the refrigerator.

### Preparation of AgNPs

**Fig. 1:**
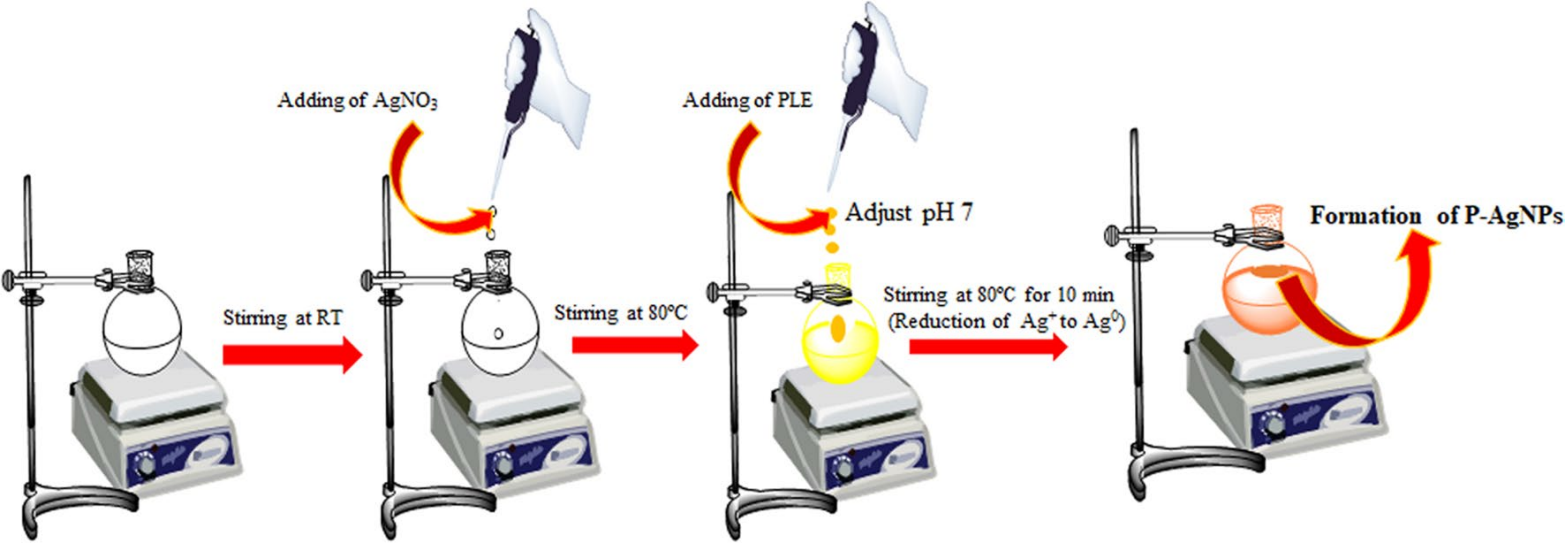
Diagram of the synthesis strategy of AgNPs

In the present study, AgNPs and AgNPs-I have been synthesized at two different temperature conditions. Briefly, in the first method, 50 mL of silver nitrate solution (0.001 M) was stirred and agitated up to 80 °C before adding different concentrations of PLE (50–1000 µL) to the AgNO_3_ solution and adjusting the pH to 7. Heating with stirring was continued till the solution of color changed from colorless to yellow which in turn indicated the formation of P − AgNPs (Fig. 1). Similarly, in the second method, AgNPs-I was obtained following the same procedure at room temperature (30 °C), excluding the exposure of high temperature. The other reaction conditions such as time, pH effect, and amount of plant extract were also optimized for the efficiency and stability of AgNPs.

### Characterizations

The absorption spectrum of AgNPs was investigated at room temperature using a quartz cell of 1.0 cm and a lab India 3000 + twin beam UV–vis spectrophotometer. High resolution transmission electron microscopy (HRTEM) on a TENAI device operating at 200 kV was used to study the exterior shape and size of AgNPs. The zeta potential of synthesized nanoparticles was determined in an aqueous solution using Zetasizer Nano ZS (Malvern Instrument, UK). Energy dispersive X-ray analysis (EDX) using an Oxford instrument INCA linked to the Field emission scanning electron microscopy (FESEM) was used to determine the elemental composition of the sample. The FT-IR spectrometer (Bruker) was used to determine the functional group involved in the fabrication of AgNPs. For the FTIR, the KBR pellet will be formed by mixing the sample with KBr at room temperature. The FT-IR spectra were then recorded over the range between 4000 and 500 cm^−1^.

In the present study, the synthesized AgNPs were evaluated for Hg^2+^ sensing abilities using colorimetric methods. Further, the developed AgNPs were also evaluated for antibacterial potential against two different bacterial strains namely *S. aureus* and *E. coli* employing in vitro disc diffusion assay.

### Colorimetric detection of Hg (II) ions

The developed AgNPs examined the abilities for the colorimetric detection of Hg^2+^ ions. Briefly, 250 µL aqueous solution of Hg^2+^ of different concentrations (0.1 to 100,000 ppb) was added to 350 µL of AgNPs solution (detection probe). The solution metal ions and silver nanoparticles are completely shaken at room temperature for about 1 min. The visual color change and the optical density were observed via UV–vis spectrophotometer.

### Fluorescence imaging

To acquire fluorescence images of apoptosis in tissue, rat liver slides were incubated with the synthesized nanoparticles (100 µg/mL) for 3 h. After that tissues were treated with 10 ppm, 100 ppm, and 1000 ppm for 40 min. Subsequently, the tissue slides were imaged using fluorescence microscopy and the excitation wavelength was fixed at 514 nm.

### Antibacterial study

#### Material and method

Both strains of bacteria were purchased from microbial type culture collection (MTCC) in Chandigarh, India. The *S. aureus* (MTCC 740) and *E. coli* (MTCC723) were used for analyzing the antibacterial activity.

#### Antibacterial activity

The antibacterial potential of PLE and AgNPs was assessed against *S. aureus* and *E. coli* strains using well diffusion and tube dilution methods.

#### Broth dilution assay

An overnight cultured bacterial inoculum (1%) was transferred to regulate the final concentration of a bacterial strain to roughly 1–5 X 10^6^ CFU/mL. Subsequently, 100µL of cultured bacterial strain was introduced to culture tubes containing 10 mL of nutritional broth media. Different concentrations of PLE and AgNPs (10–100 µg/mL) were added into the culture tubes containing media, and incubation of culture tube at 37 ºC for 24 h. Further, the absorbance was measured using a UV–vis spectrophotometer, and then the minimum inhibitory concentration (MIC), minimum bactericidal concentration (MBC), and half minimum inhibitory concentration were estimated. All these experiments were accomplished in triplicates.

#### Agar well diffusion assay

The bacterial inoculum of around 100µL was transferred to adjust the final concentration of bacterial strain to nearly 1–5 X 10^7^ CFU/mL. Consequently, bacterial culture was swabbed (20 µL) on nutrient agar plates using a sterile glass spreader and dried at room temperature. Following that, sterile micropipette tips were used to make 5 mm diameter wells in agar plates. Subsequently, these wells were filled with the PLE and AgNPs fine suspension of 10–100 ppm dissolved in dimethyl sulfoxide (1%). The plates were further incubated at 37 ºC and antibacterial activity was estimated by assessing the zone of inhibition.

### Real water analysis

To confirm the mercury sensing efficiency of the synthesized probe (AgNPs), tap water samples were collected from different sites at Banasthali Vidyapith, Banasthali, India. Water samples were collected and spiked with varying amounts of Hg^2+^ (20 ppb, 40 ppb, and 60 ppb) and analyzed by the aforementioned procedure.

## Results and discussions

The AgNPs-I was formulated at room temperature and showed characteristic broadband at 425 nm under UV–vis irradiation. The PLE aided in the reduction of Ag^+^ and stabilization of AgNPs and the time required for the synthesis of AgNPs-I was discovered to be 1 h. However, the temperature involved in the synthesis of AgNPs was at 80 °C which result in a quick reduction of Ag^+^ to Ag^0^ ions. The AgNPs synthesized by the heating method showed a narrow and stronger peak at 409 nm in UV–vis spectroscopy (Fig. S1a). The appearance of sharp spectral and blue shift of peak in UV–vis spectroscopy revealed that particles are of smaller size and in monodispersed phase. These results confirmed that the heating method is superior to the room-temperature method for the synthesis of AgNPs (Fig. S1b). Therefore, in the present study, the heating method was further employed for the preparation of AgNPs. Furthermore, the UV–vis spectra of synthesized AgNPs revealed that peak position was not change and intensity was stable even after one month (Fig. S1c). These results clearly show that the PLE is the best reducing and stabilizing agent for the synthesis of AgNPs.

### Effect of PLE, pH, and time on the synthesis of AgNPs

**Fig. 2:**
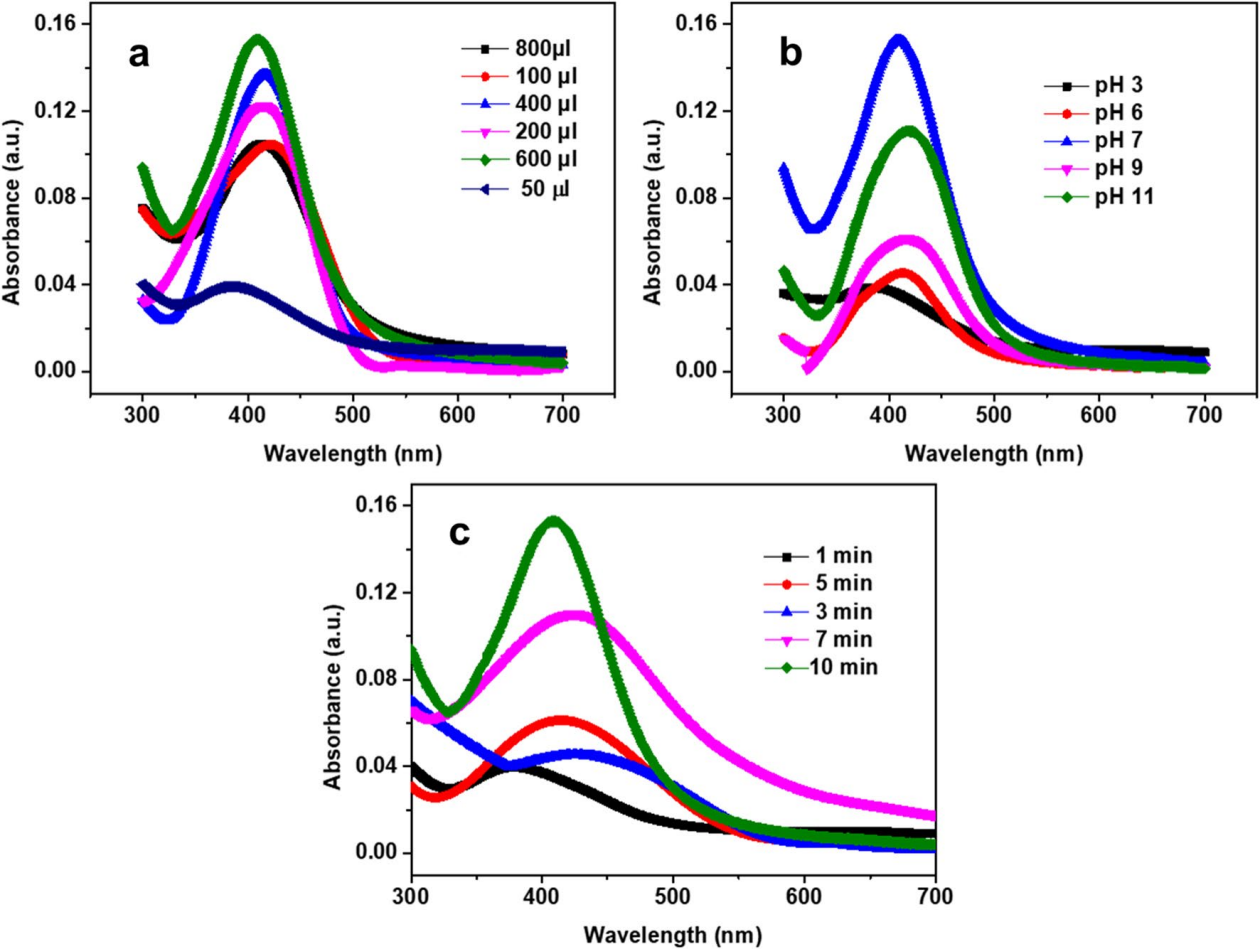
UV–Vis spectra of the synthesized AgNPs using **a** various concentrations of PLE, **b** different pH, and c different time intervals

The effect of reaction conditions on the biogenic synthesis of AgNPs were also evaluated in respect of pH, time and volume of PLE used. The different concentrations of PLE (50–800μL) were added to AgNO_3_ (0.001 M & 50 ml) solutions and alterations in SPR spectra were recorded. The intensity of the peaks was increased with an increase in the volume of PLE from 50 to 600μL (Fig. 2a). However, between the volumes from 600 to 800 μL, the color change was observed from yellow to grey with a broadening of peak and a decrease in intensity. The emergence of a secondary layer, which reduced the electron density at the thin layer of the AgNPs, most likely caused the broadening of the peak along with wavelength shift. Consequently, 600 µL of PLE was chosen as the optimal volume for the biosynthesis of AgNPs. The pH effect on nanoparticle stability was determined using SPR intensity and shift in wavelength. The color alteration of AgNPs was observed by changing the pH from 3 to 11 (Fig. 2b). The color distinction was reflected in the SPR spectra in which the intensity of the SPR band was changed in ascending manner between pH 6 to 7. The effect of different pH was resulted in change in the intensity of peak and λ. An alteration in the intensity of the SPR band was determined at extreme acidic and basic conditions which could be attributed to the destabilization of AgNPs in the solution. The optimum pH of 7.0 was chosen for the fabrication of AgNPs. One of the most important factors was reaction time, which had to be kept under control throughout the synthesis of AgNPs. The kinetics of AgNPs generation were studied using AgNO_3_ solution (0.001 M) with PLE (600 µL) under heating conditions at various time intervals to figure out the time required to complete the process. Initially, no characteristic SPR peak was observed. However, after a min, the appearance of a peak in the UV–vis spectra was visualized. This SPR peak became sharper after 10 min, which indicated the reduction of Ag^+^ ions to Ag atoms as shown in (Fig. 2c). It is noteworthy that the density of states becomes more quantized with reduction in the size of the particle and the band gap shifts to higher energies (shorter wavelengths) and this shift might be attributed formation of nanoparticles in the presence of 600 µL PLE, in10 min time interval and at pH 7.0.

### Characterization

**Fig. 3:**
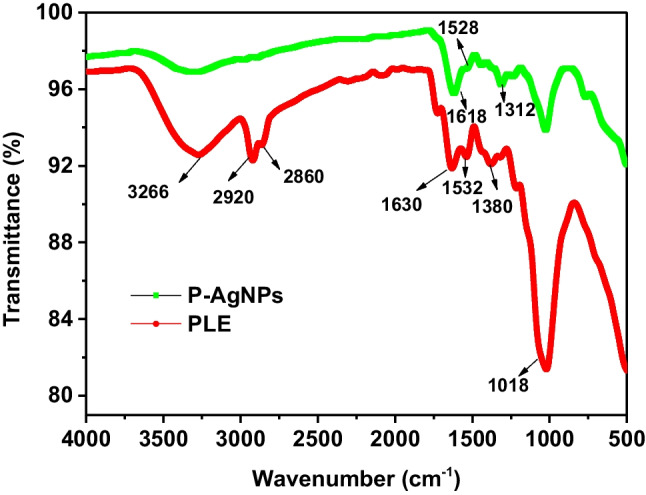
FT-IR spectra of AgNPs

The synthesized nanoparticles have been characterized by FTIR, TEM, EDX, elemental mapping, and zeta potential experiments. FT-IR spectroscopic study of AgNPs and PLE was performed to comprehend the role of PLE in the formulation and stabilization of AgNPs. Prior to the reduction of Ag^+^, the FT-IR spectrum of PLE showed distinctive peaks at 3266 cm^−1^, 2920 cm^−1^_,_ 2860 cm^−1^, 1630 cm^−1^, 1532 cm^−1^,1380 cm^−1^, 1018 cm^−1^ (G. [20]) which ascertained the presence of –OH, C–H, CH=CH_2_, and C–O stretching, respectively. FTIR spectra reveal that polyphenolic compound was present in the PLE as shown in Fig. 3.

**Fig. 4:**
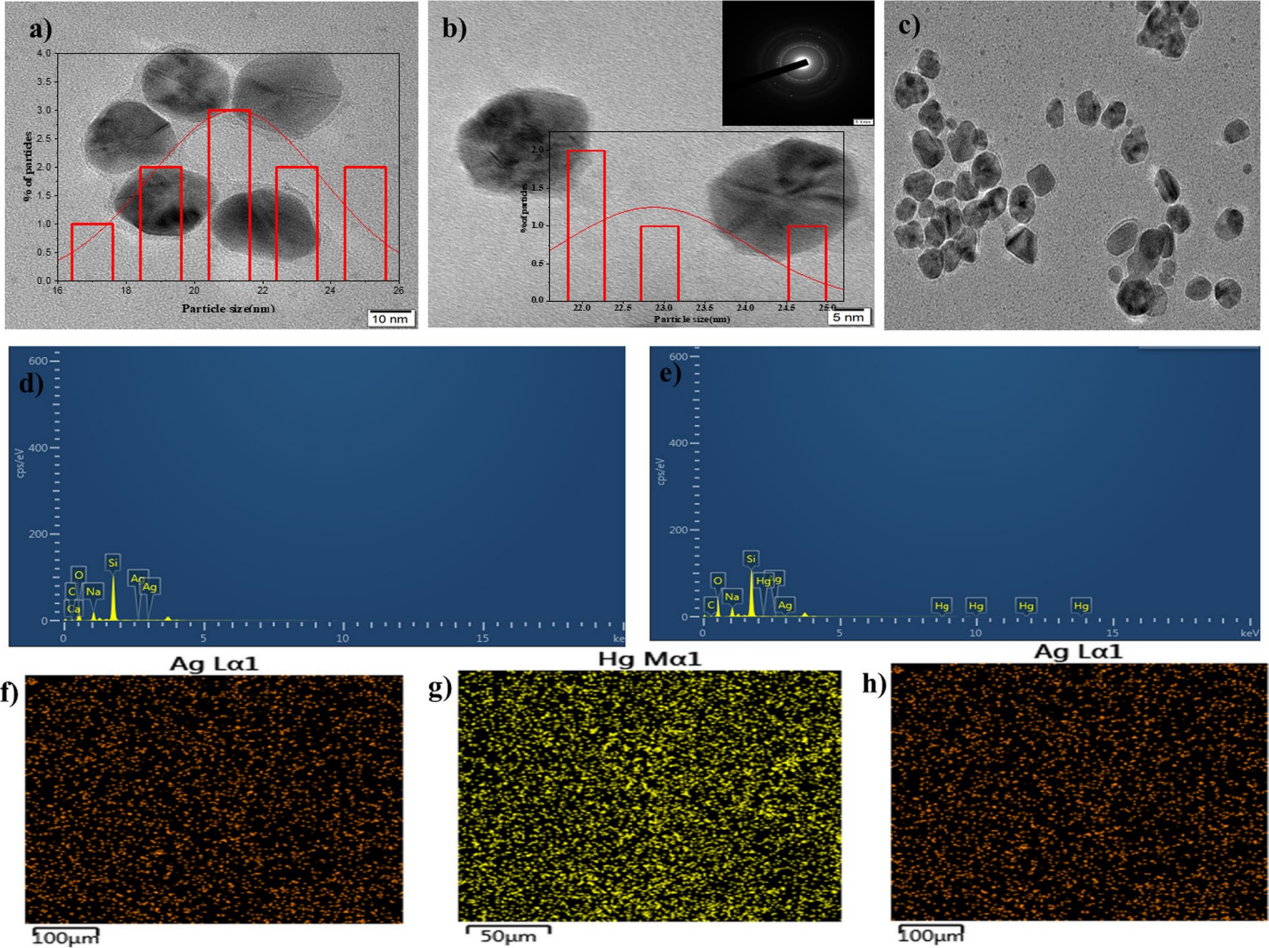
**a**–**c** TEM images of the green synthesized AgNPs and Ag-Hg(II) amalgam **b** inset correspond to SAED pattern, **c** AgNPs interaction with Hg(II) **d** Spectra of elements detected in AgNPs, **e** Ag-Hg(II) amalgam, **f** Color mapping of Ag detected during the formation of AgNPs, **g**–**h** Ag-Hg(II) amalgam in EDX analysis

The decrease in the intensity of the peak at 3266 cm^−1^ due to the reduction of Ag^+^ to Ag^0^, whereas peaks at 2920 and 2860 cm^−1^ were vanished while the minor shifting in the peaks from 1380 to 1312 cm^−1^, 1630 to 1618 cm^−1^ suggested that the phenolic compounds available in PLE acted as stabilizing and reducing agents in the synthesis of AgNPs**.** Thus, the possible mechanism for the reduction of Ag^+^ to AgNPs involves chemisorption of phenolic groups available in the PLE on the surface of Ag^0^ while the phenolic groups which are pointing away from the surface of Ag^0^ contribute towards stabilization of the AgNPs by the electrostatic repulsion [30, 40]. This result indicates that AgNPs synthesized from PLE are crystalline in nature. The TEM images depicted in (Fig. 4a–c) that synthesized AgNPs are spherical and oval in shape. The average particle size of AgNPs estimated by using Image J software, found to be between 21 and 60 nm. The crystalline structure of AgNPs was established by the presence of fringe patterns in TEM and a ring pattern in the selected area electron diffraction (SAED) (Fig. 4b inset). TEM images of AgNPs revealed a narrow size distribution of spherical particles with a size range of 21–60 nm. Figure 4c depicts the HR-TEM image of amalgam formation of Ag-Hg(II). Notably, loading of Hg^2+^on AgNPs caused amalgam formation and thus resulted an increase the particle size (Fig. 4c**).**

The elemental composition of AgNPs was evaluated through EDX analysis (Fig. 4d–e). The appearance of a silver peak and color mapping of Ag detected during the formation of AgNPs confirms the formation of AgNPs **(**Fig. 4d and f). In (Fig. 4e and g–h), the appearance of the Hg^2+^ peak confirms the interaction of Hg^2+^ metal ions with AgNPs to form Ag-Hg(II). Furthermore, the zeta potential of AgNPs was determined. The research shows a zeta potential value of − 24.4 mV as in Fig. S2. Due to electrostatic repulsion, a high negative zeta potential value specifies prolonged colloidal stability [3]. In the presence of Hg^2+^ ions, the zeta potential of AgNPs increased from − 24.4 to − 20.2 mV due to aggregation and the formation of an Ag-Hg(II) amalgam (Fig. 4g–h).

### Colorimetric detection of Hg (II) ions

#### Selectivity study of the detection probe

**Fig. 5:**
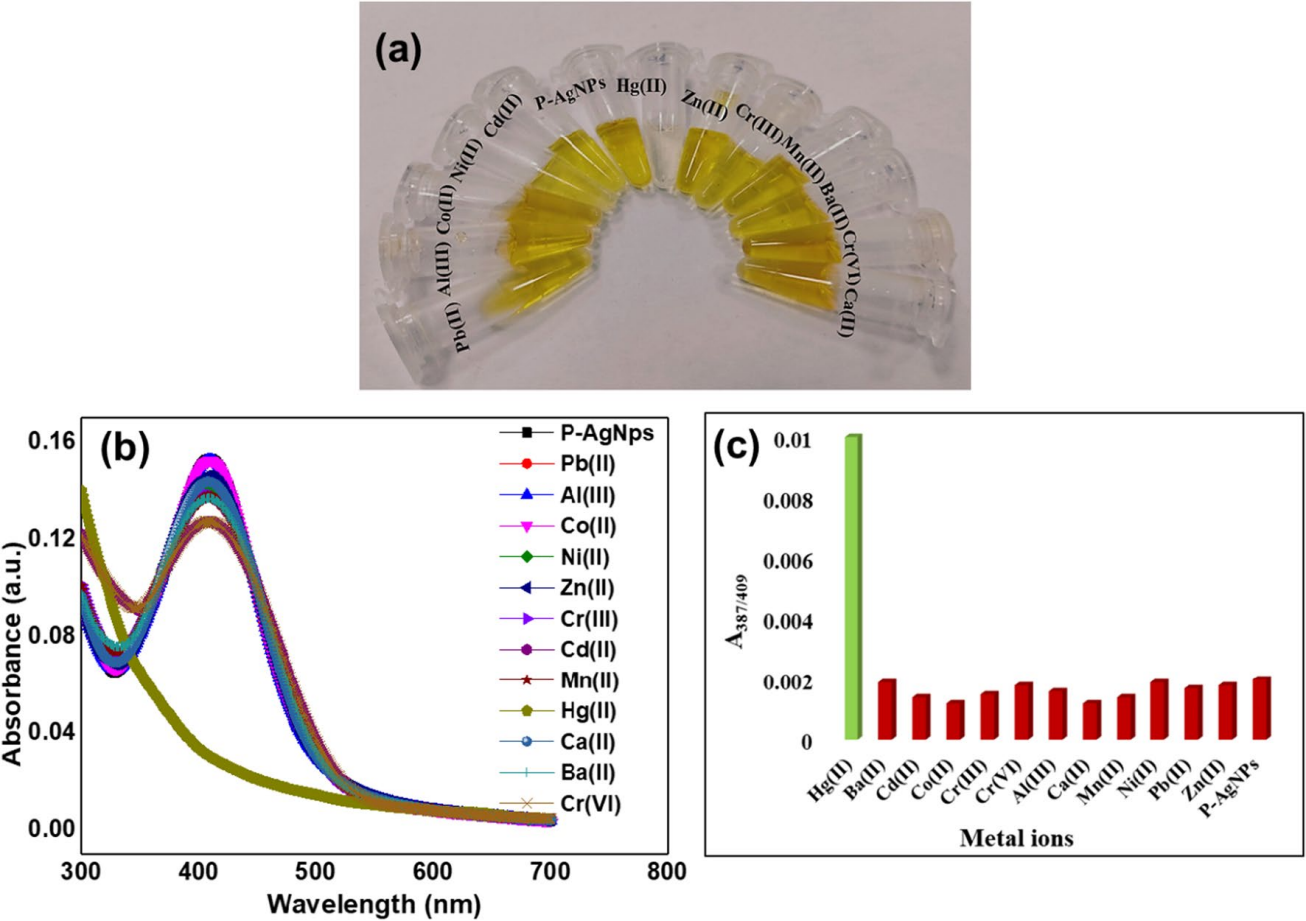
**a** The color change of AgNPs in the presence of different metal ions, **b** SPR spectra of AgNPs upon the addition of various metal ions, and **c** bar graph denotes the selectivity of AgNPs incubated with various metal ions

The optimized concentration of stabilized AgNPs was examined for their application as a colorimetric sensor. In order to evaluate the selectivity and sensitivity of AgNPs towards various metal ions, a 200 µL concentration of metal ions was added to the synthesized AgNPs. Notably, no significant change was observed in SPR spectra on the addition of various metal ion solutions (Ba^2+^, Cd^2+^, Ca^2+^, Fe^2+^, Mn^2+^, Ni^2+^, Zn^2+^, Pb^2+^, Mg^2+^, Cr^3+^, Cu^2+^) to the AgNPs solution, except for the solution containing Hg^2+^. In addition, Hg^2+^ caused a blue shift in the SPR peak with a visually detectable change from yellow color to colorless, while other metal salts showed negligible color change (Fig. 5a).

The metal ions were individually added to the mixture containing AgNPs and Hg^2+^ to assess the influence of other metal ions on the colorimetric sensing of AgNPs. The results demonstrated that the inclusion of other metal ions had no effect on the removal of the yellow color of the AgNPs solution generated by Hg^2+^ (Fig. S3). The effect of Hg^2+^ concentration (0.1 to 100,000 ppb) on the detection efficiency of AgNPs was also investigated; increasing Hg^2+^ concentrations reduced peak intensity of AgNPs, with full loss of peak intensity seen at 1000 ppm of Hg^2+^ ion. Thus, under comparable reaction conditions, produced AgNPs demonstrated very high specificity and selectivity for colorimetric detection of Hg^2+^ ion, with no sensitivity to other metal ions. Further, colorimetric responses help in monitoring the reaction condition through UV–vis spectroscopy that showed specific spectral shifts. The tested metal cations showed a slight decrease in SPR intensity, but Hg^2+^ ions showed a significantly decreased in SPR intensity and reached zero as shown in (Fig. 5b). Since the present work showed no change in extinction maxima, color and SPR intensities were detected upon the interaction of various metal ions with AgNPs except Hg^2+^, it was concluded that the synthesized probe exhibits high selectivity toward Hg^2+^ ion for colorimetric detection. The selectivity of the probe towards Hg^2+^ was further quantitatively validated by plotting the absorption intensity ratio (A_387/ 409_) of AgNPs against different types of metal cationic species (Fig. 5c). The Hg^2+^ ions exhibited a maximum increase in absorption intensity ratio (A_387/ 409_) with blue shift in peak position when compared to the other metal ions, which could be possibly due to the distinctive interaction of Hg^2+^ with AgNPs.

#### Sensitivity test for Hg (II) detection using AgNPs

**Fig. 6:**
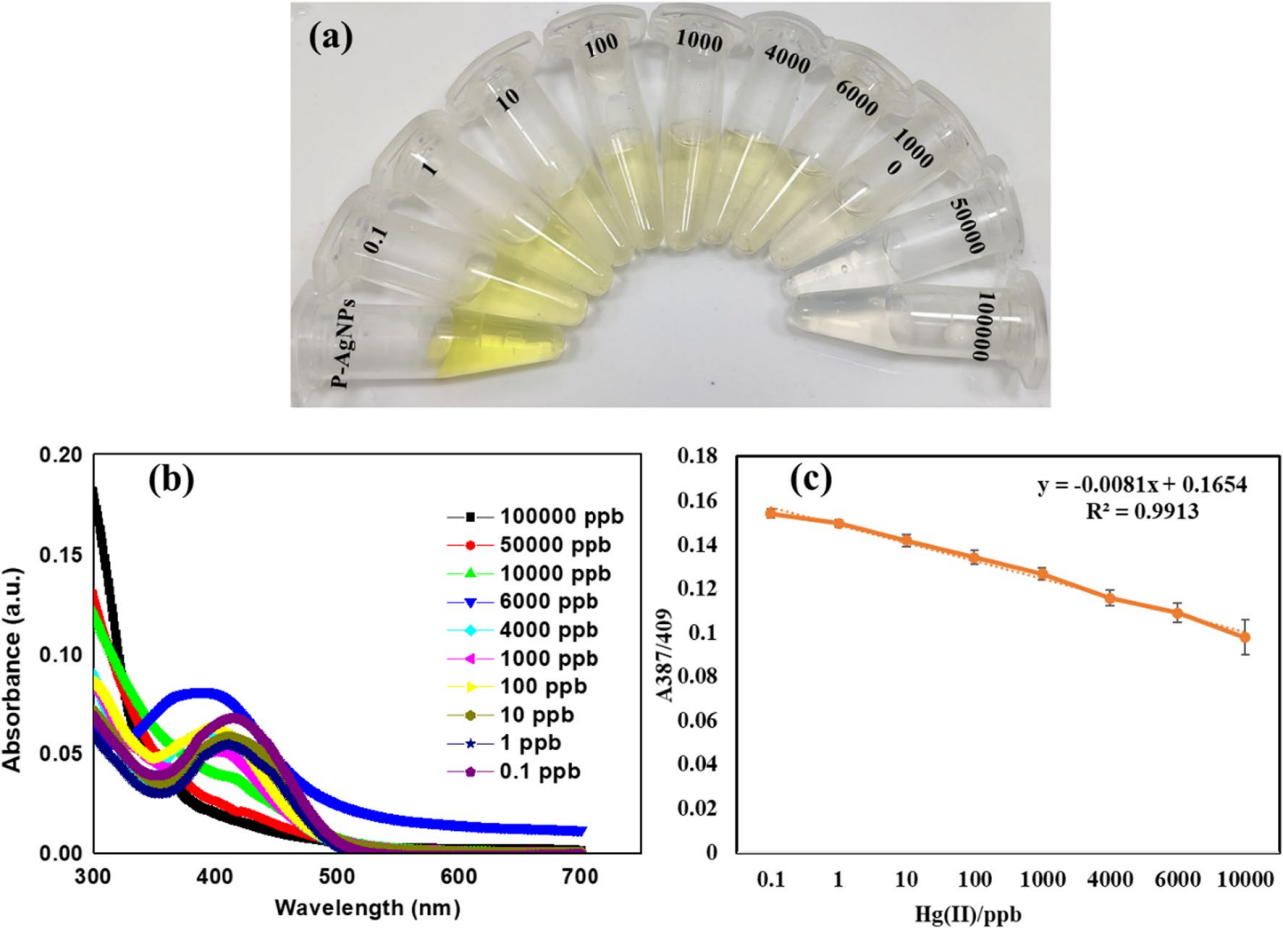
**a** Optical image of the AgNPs solution after the addition of Hg^2+^ ions in different concentrations under the optimized conditions, **b** UV–vis spectra of AgNPs at varied Hg^2+^ ion concentration, **c** Linear plot of absorbance intensity difference versus Hg^2+^ ions concentration

To estimate the lower LOD of the synthesized probe, different concentrations of Hg^2+^ ions were added to the AgNPs probe solutions. The results of the study revealed a continuous change in color with the increase in the Hg^2+^ ions concentration from 0.1 to 100,000 ppb, as shown in (Fig. 6a).

The interaction of AgNP's various concentrations of Hg^2+^ ions caused a notable change in the intensity of SPR spectra (Fig. 6b). As Hg^2+^ ions concentration increased, there was a gradual decrease in line broadening, SPR intensity, and peak shift towards shorter wavelength. (Fig. 6c) shows that there is a linear relationship (R^2^ = 0.9913) between the change in absorption intensity and Hg^2+^ ions concentrations (0.1 to 100,000 ppb). Notably, the synthesized probe's LOD was found as 0.1 ppb, making it acceptable for quantitative measurement of Hg^2+^ ions in tap water samples. LODs and limit of quantitation (LOQ) were estimated using linear plots (Fig. 6c) by applying formula 3.3*(SD of intercept/slope) and 10*(SD of intercept/slope) where SD is the standard deviation, in addition to visual LODs. From this, LODs and LOQ were found to be 4.415 ppb and 13.37 ppb for Hg^2+^, respectively.

### *Mechanism of sensing Hg*^*2*+^*ions*

**Scheme 1:**
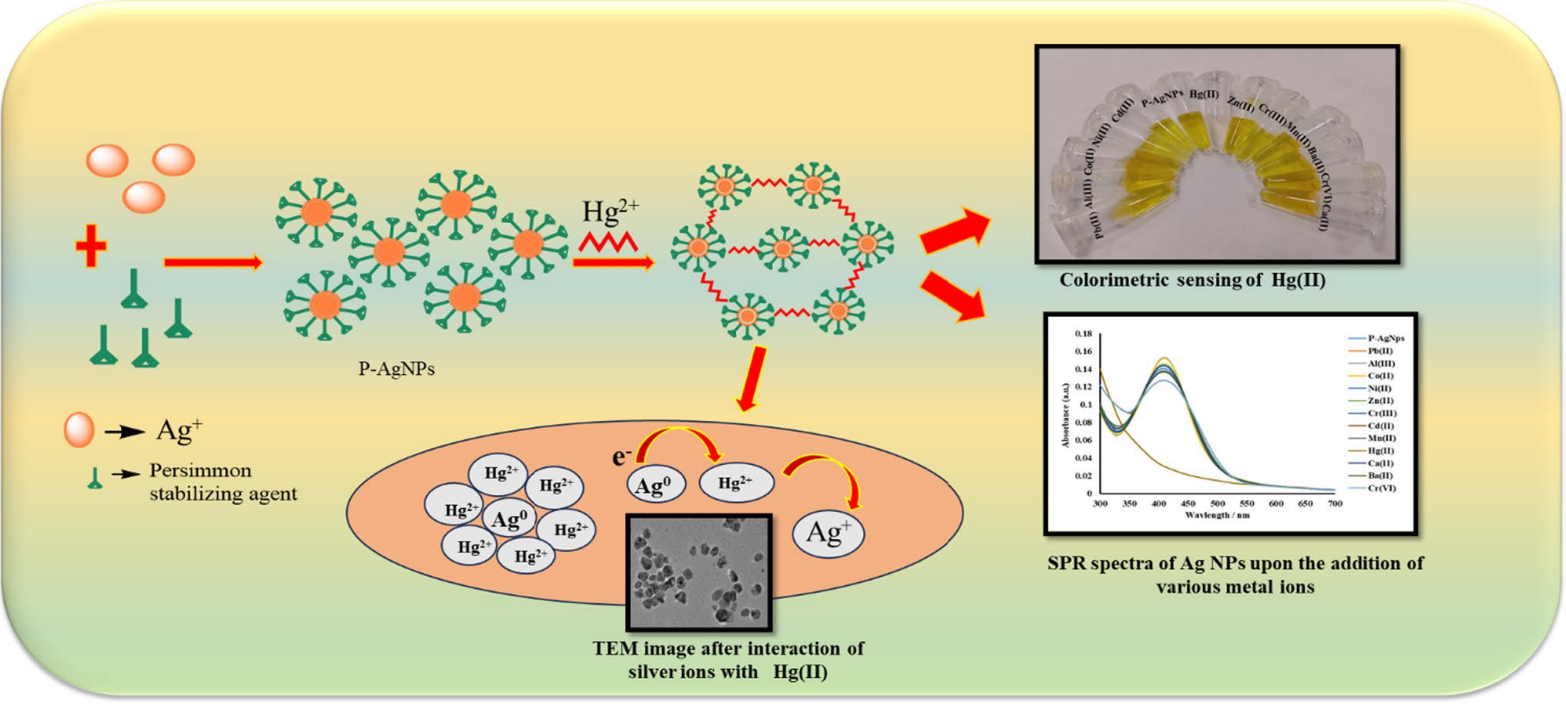
Plausible mechanisms were proposed for the interaction of AgNPs with Hg^2+^ through redox reaction mechanism

Recently, two plausible mechanisms were proposed for the interaction of AgNPs with Hg^2+^ depicted in (Scheme. 1).

The shifting of the SPR peak as well as decreasing of absorbance depends upon the interaction of synthesized nanoparticles with Hg^2+^ ions [46]. According to the first mechanism, the coating of mercury ions on the surface of AgNPs caused a reduction in absorbance and shift in the position of SPR peak.

The second plausible mechanism describes the amalgam formation between Hg^2+^ ions and AgNPs. Of note, amalgamation could be attributed to the very small electrochemical potential difference between Hg^2+^ (0.85 V) and AgNPs (0.8 V). Further, this minor difference allowed Hg^2+^ and AgNPs to interact chemically via under-potential deposition that in turn in result in amalgamation. In a study, a blue shift of the absorption peak was observed on amalgamation process when interaction of silver and gold nanoparticles with mercury took place [49]. Similarly, in the current study, a slight blue shift in the absorption peak was detected, owing to amalgam formation between Hg^2+^ and AgNPs.

Further, Saenchoopa et al. [43] described the plausible mechanism of Ag-Hg (II) amalgam formation as shown in Eq. 1. where they stated that the chemical and physical properties of AgNPs are different from the bulk metal.

1 $${\text{AgNPs }} + {\text{ Hg}}^{{{2} + }} \to {\text{Ag}}_{{{\text{n}} - {2}}} {\text{NPsHg }} + {\text{ 2Ag}}^{ + }$$

The standard electrode potential difference between silver (Ag^+^/Ag: E^0^ 0.8 V) and mercury (Hg^2+^/Hg: E^0^ 0.85 V) is so insignificant that it is unlikely to favor a faster redox reaction between Hg^2+^ and Ag^0^. However, the redox potential of nano Ag^+^/Ag in AgNPs is much distinct from that of the bulk metal Ag^+^/Ag. It is well known that a steady decline in the redox potential occurs as the size of the AgNPs decreases into the nanoscale, which reinforces the potential difference between Ag^+^/Ag nano and Hg^2+^/Hg and favors reduction of Hg^2+^ to Hg with partial oxidation of silver nanoparticles. As a result of the strong attraction between the ions of silver and mercury, Hg^2+^ is deposited on the surfaces of AgNPs, resulting in the silver-mercury amalgam, which causes substantial reduction in SPR absorption intensity and obviously, the disappearance of yellow color in AgNPs colloidal solution. This observation could be due to the nano size of the Ag ions and their strong affinity towards mercury [43].

### Fluorescence imaging

**Fig. 7:**
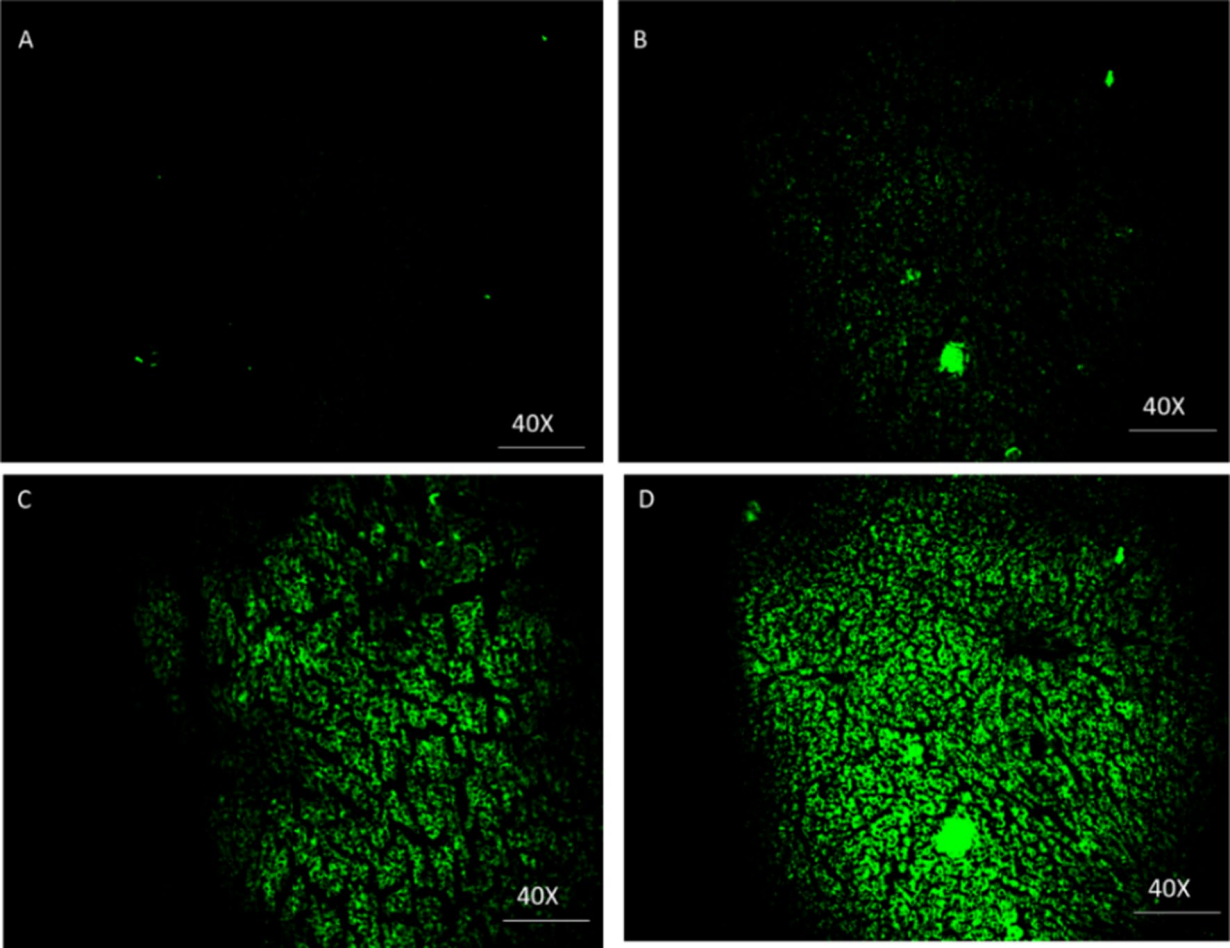
**a** Fluorescent imaging of liver tissue slides with L-AgNPs for 3 h, **b** further incubated the tissue with 10 ppm Hg^2+^for 40 min, **c** incubated with 100 ppm Hg^2+^ for 40 min, **d** incubated with 1000 ppm Hg^2+^for 40 min

The results of imaging showed that cells in liver tissue did not change after the treatment with AgNPs. These results suggested that the AgNPs could be further utilized in imaging in liver tissue slides due to their good biocompatibility. Subsequently, a fluorescence microscope was used for the imaging of Hg^2+^ in liver tissues immediately. As shown in Fig. 7a, a blank slide with very weak green fluorescence could be observed in the liver tissue (Ex = 514 nm) after the introduction of AgNPs into slides. When slides were treated with Hg^2+^ (10 ppm) for a further 40 min, the slide showed a very weak fluorescence (Fig. 7b). However, when 100 and 1000 ppm Hg^2+^ were added into the above tissue slides loaded with Hg^2+^ for 40 min, outstanding green fluorescence was observed (Fig. 7c and d). As illustrated in Fig. 7b-d, the liver tissue cells remained as expected, and AgNPs could be used to sense Hg^2+^ in liver tissues.

### Antibacterial activity

The antibacterial activity of PLE and AgNPs was analyzed against gram positive and negative strains of bacteria using the in vitro agar well diffusion method and broth dilution method. A well-defined zone of inhibitions was observed for PLE and AgNPs against *E. coli* and *S. aureus* in agar well diffusion method. At 100 µg/mL, the zone of inhibition for PLE was found as 1.63 ± 0.15 and 0.92 ± 0.01 and 4.6 ± 0.2 and 3.63 ± 0.20 for AgNPs against *E. coli* and *S. aureus* respectively that in turn indicated the synthesized AgNPs exhibited better antibacterial activity against both the bacterial strains, when compared to PLE (Table 1).

**Table 1:** Zone of inhibition of PLE and AgNPs against *E. coli* and* S. aureus*

Concentration (µg/mL)	*E. coli*		*S. aureus*	
	PLE	AgNPs	PLE	AgNPs
10	1.33 ± 0.11	2.6 ± 0.2	0.46 ± 0.01	2.23 ± 0.15
50	1.6 ± 0.2	3.6 ± 0.2	0.56 ± 0.02	2.66 ± 0.05
100	1.63 ± 0.15	4.6 ± 0.2	0.92 ± 0.01	3.63 ± 0.20

**Fig. 8:**
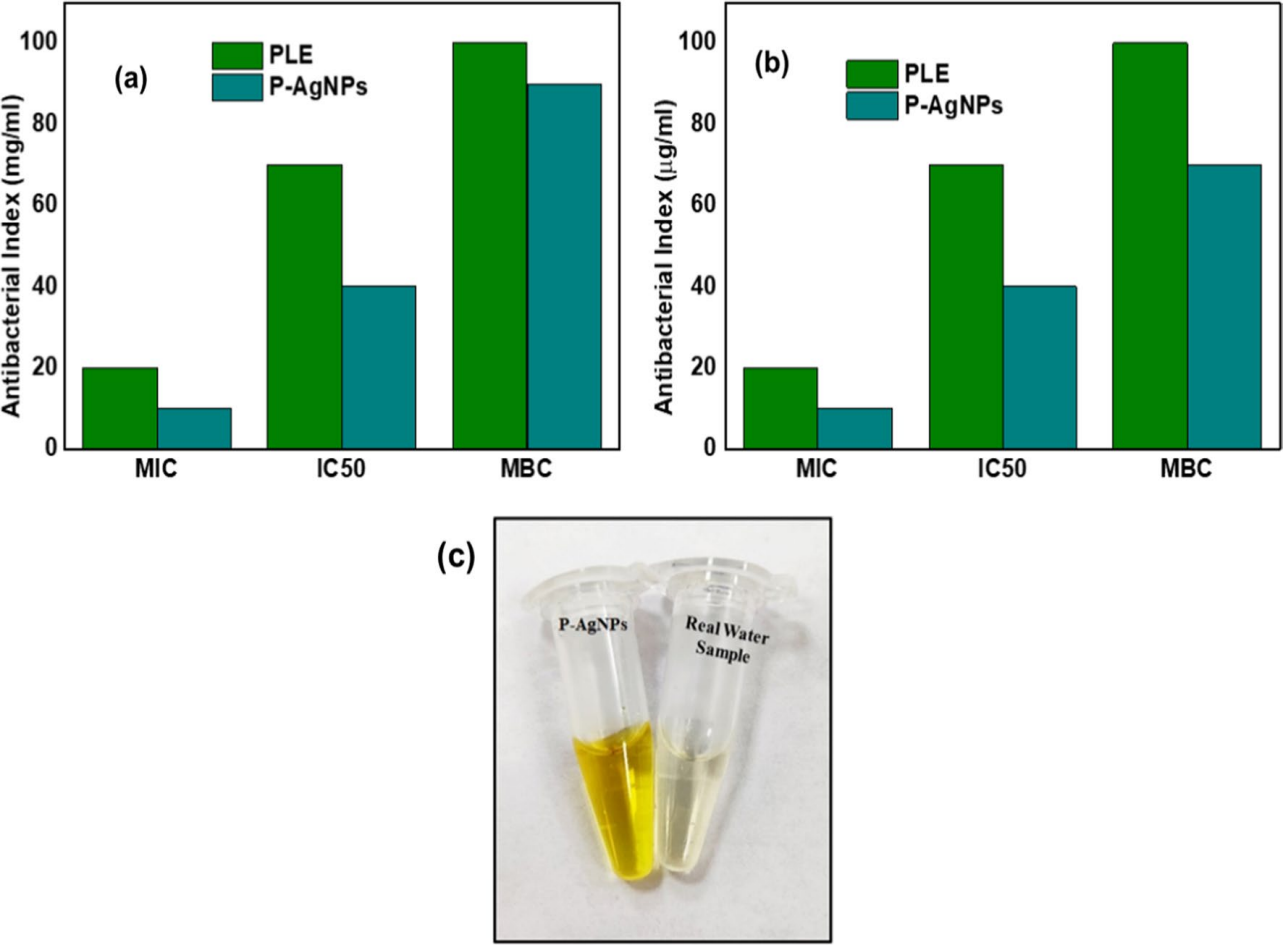
The effect of PLE and AgNPs on IC_50_, MIC and MBC against **a** *E. coli*; **b** *S. aureus*; (**c**) AgNPs colorimetric response as the function of Hg^2+^ ion concentration in spiked tap water samples

In the broth dilution method, the MIC for PLE and AgNPs against *S. aureus* and *E. coli was* at 20 and 10 µg/mL, respectively. In addition, the MBC for PLE against *E. coli* and *S. aureus* was 100 µg/mL, and for AgNPs against *S. aureus* and *E. coli* was 80 and 90 µg/mL, respectively. Notably, AgNPs exhibited an IC_50_ of 40 µg/mL against *E. coli* and *S. aureus* as shown in Fig. 8.

The AgNPs exhibited significant antibacterial activity than PLE because of their smaller size, larger specific surface area, and spherical shape. Based on the surface area available for the interaction, AgNPs can bind bacteria, and respiratory and permeability function has been disturbed. Smaller particles exhibit larger surface areas which have a greater ability to penetrate bacterial cell membranes which results in a significant bactericidal effect than the larger particles [9]. Thus, the antibacterial activity of synthesized AgNPs is dependent on the morphology and size of nanoparticles. The inverse relationship exists between the size of AgNPs and potential antibacterial activity. The synthesized AgNPs act as effective antibacterial drugs for various biomedical applications as well as provide fast and sensitive responses towards the Hg^2+^ ions.

### Real water analysis

The synthesized colorimetric probe was examined for sensing the Hg^2+^ ions in the real water samples. The initial colorimetric test revealed that a detectable amount of mercury ion was not found in the real water sample, so water is spiked with 4 mL of 20, 40, and 60 ppb Hg^2+^ ions. The mercury ion can be detected by colorimetric assay without being affected by other interfering metal ions. The validity of the method was further analyzed by a recovery test for the determination of Hg^2+^ in tap water samples. The mercury was spiked with three different concentrations and then examined using the standard addition and AAS method. The recoveries of the Hg^2+^ ions were found to be more than 99% in all the samples and the results were consistent in both the methods. Table 2 result confirmed that synthesized AgNPs could be used as portable and cheap colorimetric sensors for the detection of Hg^2+^ ions in real water analysis (Fig. 8c). Furthermore, we compared the colorimetric sensor synthesized in the present study with the other reported nanoparticles as a sensor. The easy formulation, fast, sensitive response time, biological application and development of cheap probes/sensors make the present work superior to the other described methods in the literature.

**Table 2:** The recovery results from spiked tap water samples (n = 3)

Sample	Hg^2+^ (ppb)			Recovery proposed method (%)	Recovery AAS method (%)
	Added	Proposed method	AAS method		
Tap water	20	19.81 ± 0.12	19.91 ± 0.025	99.06	99.56
	40	39.22 ± 0.04	39.86 ± 0.072	99.06	99.56
	60	60.05 ± 0.94	60.80 ± 0.43	99.06	99.56

Recovery (%) = 100 × (C_found_/C_added_)

### Merits of the sensor probe

The properties of different biosensor reported in the literature for the detection of Hg^2+^ in water is summarized in Table 3. These sensors have been compared on the basis of their LOD value and procedure of synthesis. The Hg^2+^ sensor was developed using two plant extracts with a LOD value of 2.2 µM. The use of two extracts and lower sensitivity, call the sensor's feasibility into question [18]. Similarly, the sensor developed by using *Asystasia gangetica* leaf may be considered as most sensitive with a high LOD value of 3.25 nM but it requires a konjac glucomannan (KgM) solution as a stabilizer [23]. Likewise, in other cases where AgNPs based Hg^2+^ sensors were developed using simple synthetic procedures but the sensitivity of these sensors is very low (indicated by higher LOD value) [4, 8, 18, 23, 39, 48]. In comparison to these developed sensors, the synthesized AgNPs exhibited higher sensitivity with a lower LOD value of 0.1 ppb, with excellent selectivity for Hg^2+^, and can be effectively utilized in diluted aqueous media to address water contaminated with Hg^2+^.

**Table 3:** Comparative data based on the green synthesis of AgNPs showing colorimetric sensing of Hg^2+^ using UV–vis spectroscopy

S.No	Sensor	Detection method	LOD	References
1	Manna of Hedysarum and Soap-root plant extract	Colorimetric	2.20 µM	[18]
2	Asystasia gangetica and konjac glucomannan (KgM) as a stabilizer	Colorimetric	3.25 nM	[23]
3	Phyllanthus acidus extract	Colorimetric	0.80 µM	[48]
4	*Citrus japonica* leaves extract	Colorimetric	0.09 µM	[8]
5	Carrageenan extract	Colorimetric	1 µM	[39]
6	Orange Peel	Colorimetric	0.25 µM	[4]
7	Chlorophyll	Colorimetric	2.7 µM	[13]
8	Kokum fruit	Colorimetric	8.7 ppb	[47]
9	PLE	Colorimetric	0.1 ppb	Present work

## Conclusions

In the present report, an easy, efficient, and economical method has been reported for the biogenic synthesis of silver nanoparticles from persimmon leaves. The persimmon leaves constituent various metabolites for the bioactive reduction of nanoparticles. Several characterization techniques, including FTIR, UV–vis, TEM, and zeta potential, demonstrated the successful synthesis of AgNPs particles. The interference study demonstrated that other cations have no effect on Hg^2+^ sensing of AgNPs. The spiked-recovery of Hg^2+^ in tap water samples revealed notable accuracy with lower LOD values. Synthesized AgNPs could be used as portable and cheap colorimetric sensors for the detection of Hg^2+^ ions in real water analysis and could be used for fluorescent imaging of Hg^2+^ in liver tissues Further, AgNPs showed antibacterial potential against most common bacterial pathogens. To the best of our knowledge, this is the first ever sensor that has outstanding sensitivity up to 0.1 ppb level for Hg^2+^ in aqueous medium and could be explored in water remediation.

### Supplementary Information

**Additional file 1.**

## Data Availability

The data generated during the current study are available from the corresponding author on reasonable request.

## References

[1] Ahmed F, Kabir H, Xiong H (2020). Dual colorimetric sensor for Hg^2+^/Pb^2+^ and an efficient catalyst based on silver nanoparticles mediating by the root extract of *Bistorta amplexicaulis*. Front Chem.

[2] Ahmed KBA, Senthilnathan R, Megarajan S, Anbazhagan V (2015). Sunlight mediated synthesis of silver nanoparticles using redox phytoprotein and their application in catalysis and colorimetric mercury sensing. J Photochem Photobiol B Biol.

[3] Alam MN, Chatterjee A, Das S (2015). Burmese grape fruit juice can trigger the “logic gate”-like colorimetric sensing behavior of Ag nanoparticles towards toxic metal ions. RSC Adv.

[4] Aminu A, Oladepo SA (2021). Fast orange peel-mediated synthesis of silver nanoparticles and use as visual colorimetric sensor in the selective detection of mercury(II) Ions. Arab J Sci Eng.

[5] Annadhasan M, Muthukumarasamyvel T, Sankar Babu VR, Rajendiran N (2014). Green synthesized silver and gold nanoparticles for colorimetric detection of Hg^2+^, Pb^2+^, and Mn^2+^ in aqueous medium. ACS Sustain Chem Eng.

[6] Balasurya S, Ahmad P, Thomas AM (2020). Rapid colorimetric and spectroscopy based sensing of mercury by surface functionalized silver nanoparticles in the presence of tyrosine. Opt Commun.

[7] Balasurya S, Syed A, Thomas AM (2020). Rapid colorimetric detection of mercury using silver nanoparticles in the presence of methionine. Spectrochim Acta Part A Mol Biomol Spectrosc.

[8] Bhagat S, Shaikh H, Nafady A (2022). Trace level colorimetric Hg^2+^ sensor driven by citrus japonica leaf extract derived silver nanoparticles: green synthesis and application. J Clust Sci.

[9] Bindhu MR, Umadevi M (2014). Surface plasmon resonance optical sensor and antibacterial activities of biosynthesized silver nanoparticles. Spectrochim Acta Part A Mol Biomol Spectrosc.

[10] Chen L, Li J, Chen L (2014). Colorimetric detection of mercury species based on functionalized gold nanoparticles. ACS Appl Mater Interfaces.

[11] Chen GH, Chen WY, Yen YC, Wang CW, Chang HT, Chen CF (2014). Detection of mercury (II) ions using colorimetric gold nanoparticles on paper-based analytical devices. Anal Chem.

[12] Choudhary MK, Garg S, Kaur A (2020). Green biomimetic silver nanoparticles as invigorated colorimetric probe for Hg^2+^ ions: a cleaner approach towards recognition of heavy metal ions in aqueous media. Mater Chem Phys.

[13] Demirezen Yılmaz D, Aksu Demirezen D, Mıhçıokur H (2021). Colorimetric detection of mercury ion using chlorophyll functionalized green silver nanoparticles in aqueous medium. Surf Interfaces.

[14] Elgendy K, Saad MZ, Amer A (2023). Eco-friendly method for removal of some heavy metals from water by persimmon leaves ash and its nano adsorbent: analytical and physical study. Egypt J Chem.

[15] Ertürk AS (2019). Biosynthesis of silver nanoparticles using *Epilobium parviflorum* green tea extract: analytical applications to colorimetric detection of Hg^2+^ ions and reduction of hazardous organic dyes. J Clust Sci.

[16] Faghiri F, Ghorbani F (2019). Colorimetric and naked eye detection of trace Hg^2+^ ions in the environmental water samples based on plasmonic response of sodium alginate impregnated by silver nanoparticles. J Hazard Mater.

[17] Faghiri F, Ghorbani F (2020). Synthesis of graphene oxide nanosheets from sugar beet bagasse and its application for colorimetric and naked eye detection of trace Hg^2+^ in the environmental water samples. Microchem J.

[18] Farhadi K, Forough M, Molaei R (2012). Highly selective Hg^2+^ colorimetric sensor using green synthesized and unmodified silver nanoparticles. Sensors Actuators B Chem.

[19] Firdaus ML, Aprian A, Meileza N (2019). Smartphone coupled with a paper-based colorimetric device for sensitive and portable mercury ion sensing. Chemosensors.

[20] Narasimha G, Raju BDP (2011). Mushrooms (*Agaricus bisporus*) mediated biosynthesis of sliver nanoparticles, characterization and their antimicrobial activity. Int J Nano Dimens.

[21] Ghosh S, Mondal A (2020). Aggregation chemistry of green silver nanoparticles for sensing of Hg^2+^ and Cd^2+^ ions. Coll Surf A Physicochem Eng Asp.

[22] Janani B, Syed A, Thomas AM (2020). UV–vis spectroscopic method for the sensitive and selective detection of mercury by silver nanoparticles in presence of alanine. Optik.

[23] Jayeoye TJ, Eze FN, Olatunji OJ, Tyopine AA (2022). Synthesis of biocompatible Konjac glucomannan stabilized silver nanoparticles, with *Asystasia gangetica* phenolic extract for colorimetric detection of mercury (II) ion. Sci Rep.

[24] Jayeoye TJ, Sirimahachai U, Wattanasin P, Rujiralai T (2022). Eco-friendly poly(aniline boronic acid)/gum tragacanth stabilized silver nanoparticles nanocomposite for selective sensing of Hg2+. Microchem J.

[25] Kalam A, Al-Sehemi AG, Alrumman S (2018). Colorimetric optical chemosensor of toxic metal ion (Hg^2+^) and biological activity using green synthesized AgNPs. Green Chem Lett Rev.

[26] Kateshiya MR, George G, Rohit JV (2020). Ractopamine as a novel reagent for the fabrication of gold nanoparticles: colorimetric sensing of cysteine and Hg^2+^ ion with different spectral characteristics. Microchem J.

[27] Kumar KS, Ramakrishnappa T (2021). Green synthesized uncapped Ag colloidal nanoparticles for selective colorimetric sensing of divalent Hg and H_2_O_2_. J Environ Chem Eng.

[28] Kumar V, Singh DK, Mohan S (2017). Green synthesis of silver nanoparticle for the selective and sensitive colorimetric detection of mercury (II) ion. J Photochem Photobiol B Biol.

[29] Kumar VV, Anthony SP (2016). Highly selective colorimetric sensing of Hg^2+^ ions by label free AuNPs in aqueous medium across wide pH range. Sensors Actuators B Chem.

[30] Kumari S, Sharma KS, Nemiwal M (2022). Simultaneous detection of aqueous aluminum(III) and chromium(III) using Persea americana reduced and capped silver nanoparticles. Int J Phytoremediation.

[31] Lee S-Y, Choi H-J (2018). Persimmon leaf bio-waste for adsorptive removal of heavy metals from aqueous solution. J Environ Manag.

[32] Li H, Wang W, Wang Z (2021). Analyte-enhanced photocatalytic activity of CdSe/ZnS quantum dots for paper-based colorimetric sensing of Hg^2+^ under visible light. Microchem J.

[33] Manasfi T (2021). Ozonation in drinking water treatment: an overview of general and practical aspects, mechanisms, kinetics, and byproduct formation. Compr Anal Chem.

[34] Marimuthu V, Chandirasekar S, Rajendiran N (2018). Green synthesis of sodium cholate stabilized silver nanoparticles: an effective colorimetric sensor for Hg^2+^ and Pb^2+^ ions. ChemistrySelect.

[35] Memon R, Memon AA (2022). Ultrasensitive colorimetric detection of Hg^2+^ in aqueous media via green synthesis by *Ziziphus mauritiana* Leaf extract-based silver nanoparticles. Int J Environ Anal Chem.

[36] Mortazavi-Derazkola S, Hosseinzadeh M, Yousefinia A, Naghizadeh A (2021). Green synthesis and investigation of antibacterial activity of silver nanoparticles using eryngium bungei boiss plant extract. J Polym Environ.

[37] Mortazavi-Derazkola S, Yousefinia A, Naghizadeh A (2021). Green synthesis and characterization of silver nanoparticles using elaeagnus angustifolia bark extract and study of its antibacterial effect. J Polym Environ.

[38] Musikavanhu B, Muthusamy S, Zhu D (2022). A simple quinoline-thiophene Schiff base turn-off chemosensor for Hg^2+^ detection: spectroscopy, sensing properties and applications. Spectrochim Acta Part A Mol Biomol Spectrosc.

[39] Narayanan KB, Han SS (2017). Highly selective and quantitative colorimetric detection of mercury(II) ions by carrageenan-functionalized Ag/AgCl nanoparticles. Carbohydr Polym.

[40] Oluwafemi OS, Anyik JL, Zikalala NE, Sakho EHM (2019). Biosynthesis of silver nanoparticles from water hyacinth plant leaves extract for colorimetric sensing of heavy metals. Nano-Struct Nano-Objects.

[41] Preman NK, Jain S, Antony A, Shetty DM, Fathima N, Prasad KS, Johnson RP (2023). Stimuli-responsive copolymer-mediated synthesis of gold nanoparticles for nanozyme-based colorimetric detection of mercury (II) ions. ACS Appl Polym Mater.

[42] Sadalage PS, Patil RV, Padvi MN, Pawar KD (2020). Almond skin extract mediated optimally biosynthesized antibacterial silver nanoparticles enable selective and sensitive colorimetric detection of Fe^+2^ ions. Coll Surf B Biointerfaces.

[43] Saenchoopa A, Boonta W, Talodthaisong C (2021). Colorimetric detection of Hg(II) by γ-aminobutyric acid-silver nanoparticles in water and the assessment of antibacterial activities. Spectrochim Acta Part A Mol Biomol Spectrosc.

[44] Sahu D, Sahoo G, Mohapatra P, Swain SK (2019). Dual activities of nano silver embedded reduced graphene oxide using clove leaf extracts: Hg^2+^ sensing and catalytic degradation. ChemistrySelect.

[45] Samari F, Salehipoor H, Eftekhar E, Yousefinejad S (2018). Low-temperature biosynthesis of silver nanoparticles using mango leaf extract: catalytic effect, antioxidant properties, anticancer activity and application for colorimetric sensing. New J Chem.

[46] Sangaonkar GM, Desai MP, Dongale TD, Pawar KD (2020). Selective interaction between phytomediated anionic silver nanoparticles and mercury leading to amalgam formation enables highly sensitive, colorimetric and memristor-based detection of mercury. Sci Rep.

[47] Sangaonkar GM, Desai MP, Dongale TD, Pawar KD (2020). Selective interaction between phytomediated anionic silver nanoparticles and mercury leading to amalgam formation enables highly sensitive, colorimetric and memristor-based detection of mercury. Sci Rep.

[48] Sk I, Khan MA, Ghosh S (2019). A reversible biocompatible silver nanoconstracts for selective sensing of mercury ions combined with antimicrobial activity studies. Nano-Struct Nano-Objects.

[49] Tanvir F, Yaqub A, Tanvir S (2019). Colorimetric detection of mercury ions in water with capped silver nanoprisms. Materials.

[50] Vinod Kumar V, Anbarasan S, Christena LR (2014). Bio-functionalized silver nanoparticles for selective colorimetric sensing of toxic metal ions and antimicrobial studies. Spectrochim Acta Part A Mol Biomol Spectrosc.

[51] Wang J, Wu J, Zhang Y (2021). Colorimetric and SERS dual-mode sensing of mercury (II) based on controllable etching of Au@Ag core/shell nanoparticles. Sensors Actuators B Chem.

[52] Xie C, Xie Z, Xu X, Yang D (2015). Persimmon (*Diospyros kaki L*.) leaves: a review on traditional uses, phytochemistry and pharmacological properties. J Ethnopharmacol.

[53] Yang YK, Yook KJ, Tae J (2005). A rhodamine-based fluorescent and colorimetric chemodosimeter for the rapid detection of Hg^2+^ ions in aqueous media. J Am Chem Soc.

