# Supplementary material for: Green synthesized AgNPs as a probe for colorimetric detection of Hg (II) ions in aqueous medium and fluorescent imaging in liver cell lines and its antibacterial activity
Source: Discov Nano. 2024 May 2;19(1):78. doi: 10.1186/s11671-024-04014-8 (PMC11065856; doi:10.1186/s11671-024-04014-8)
Supplement: Supplementary file 1 — Additional file 1. [file 11671_2024_4014_MOESM1_ESM.docx]

**Supporting information**

**Green Synthesized AgNPs as a Probe for Colorimetric Detection of Hg (II) Ions in Aqueous Medium and Fluorescent Imaging in Liver Cell Lines and its Antibacterial Activity**

Sanjana Tewari^†, 1^, Shalini Sahani^†, 2^, Neetu Yaduvanshi^1^, Ritu Painuli^3^, Nalini Sankararamakrishnan^4^, Jaya Dwivedi^1,^ *, Swapnil Sharma^5^, Sung Soo Han^2,^ *

^1^Department of Chemistry, Banasthali Vidyapith, Banasthali, Rajasthan-304 022, India

^2^School of Chemical Engineering, Yeungnam University, 280 Daehak-ro, Gyeongsan, 38541, South Korea

^3^Department of Chemistry, School of Applied and Life Sciences, Uttaranchal University, Dehradun, Uttarakhand, 248007, India

^4^Centre for Environmental Science and Engineering, Indian Institute of Technology, Kanpur, Uttar Pradesh, 208016, India

^5^Department of Pharmacy, Banasthali Vidyapith, Banasthali, Rajasthan, 304022, India

***Corresponding Author (s):**

[**Email:** jayadwivedi@yahoo.co.in](mailto:Email:%20jayadwivedi@yahoo.co.in) (Jaya Dwivedi), [sshan@yu.ac.kr](mailto:sshan@yu.ac.kr) (Sung Soo Han).

^†^ These authors contribute equally.


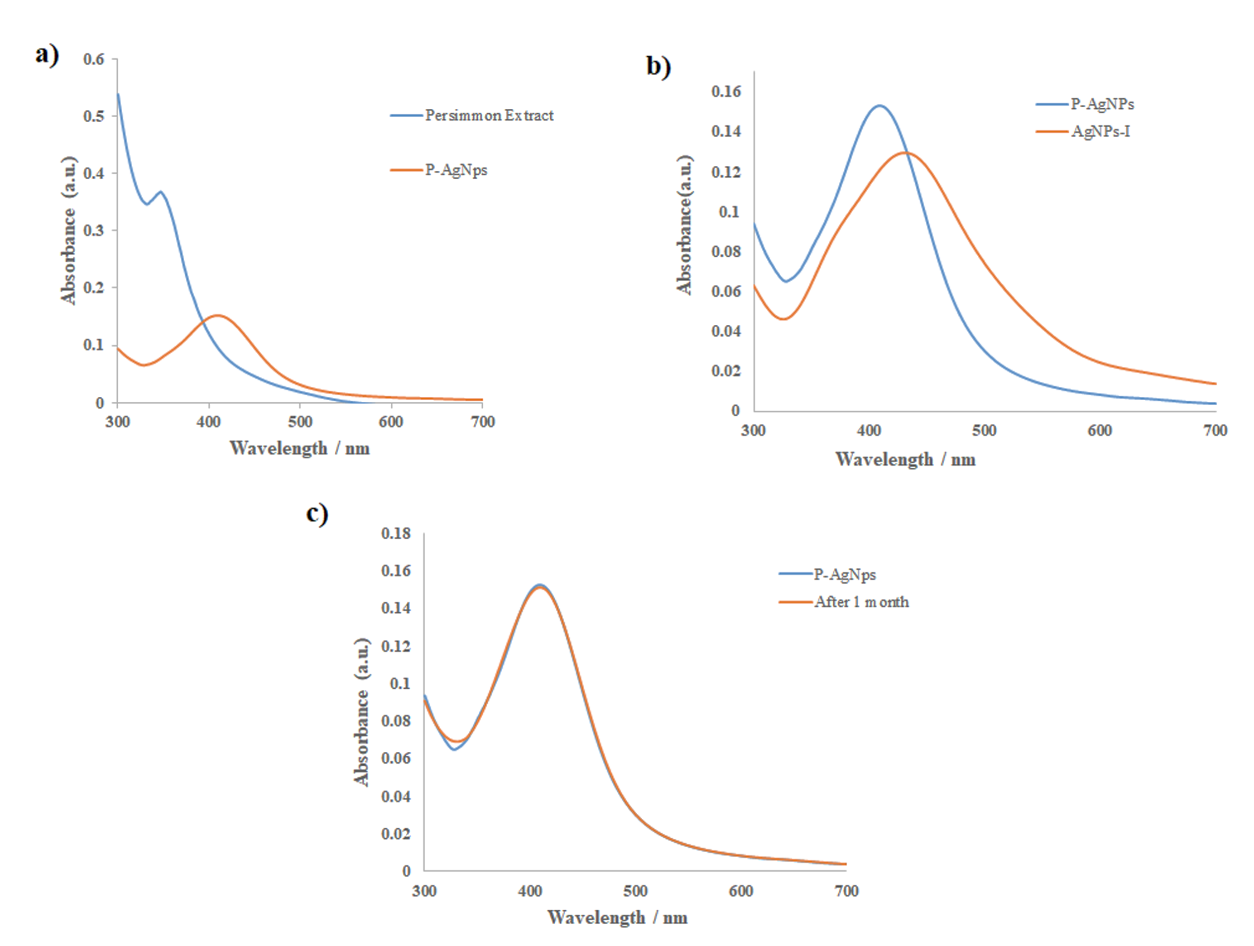


**Fig. S1.** UV-Vis spectra a) fresh Persimmon extract and P-AgNPs, (b) Synthesis of P-AgNPs by the heating method and at room temperature (c) stability of P-AgNPs after one month.


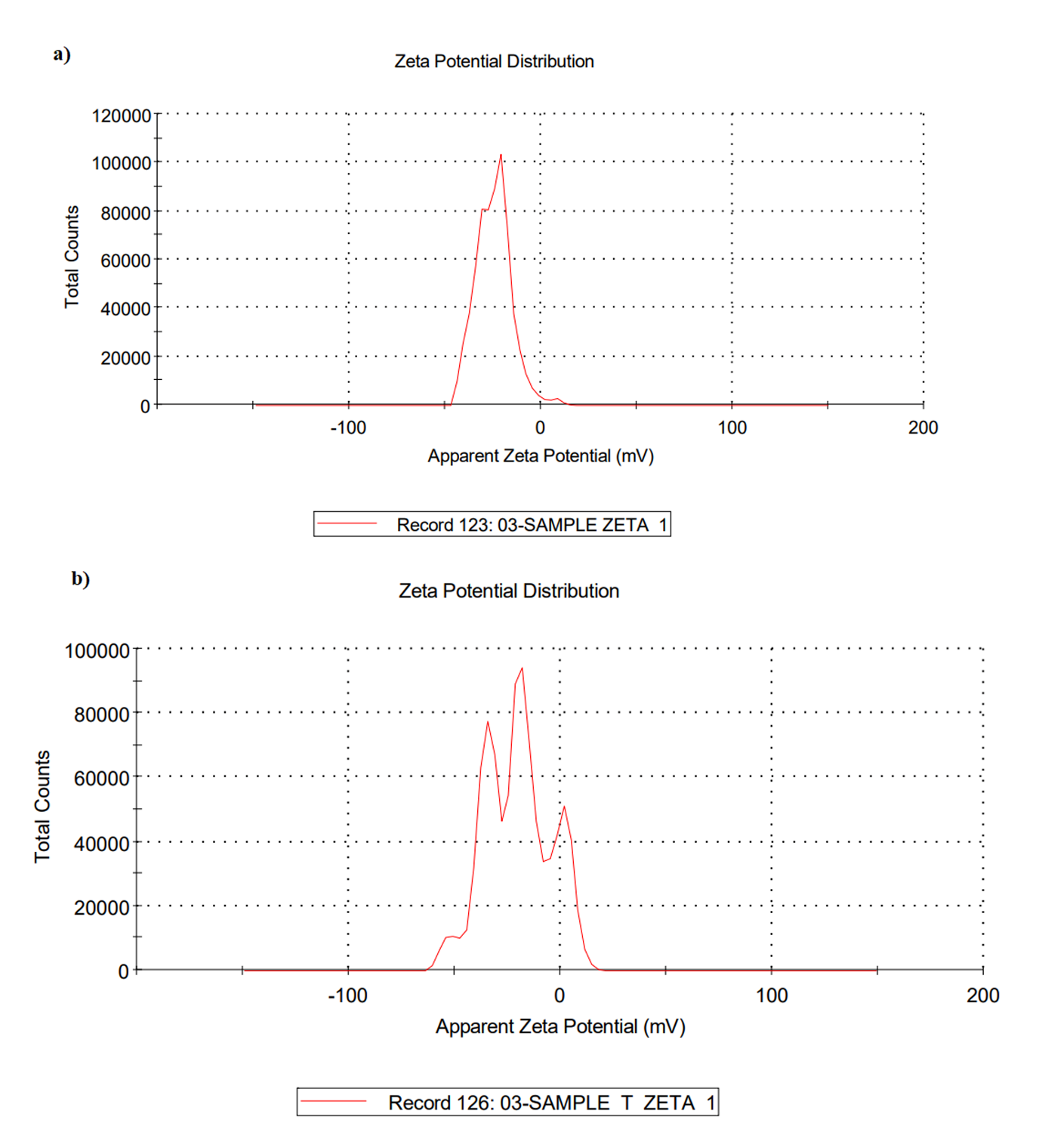


**Fig. S2.** Zeta potential distribution of (a) P-AgNPs, (b) (P-AgNPs + Hg)


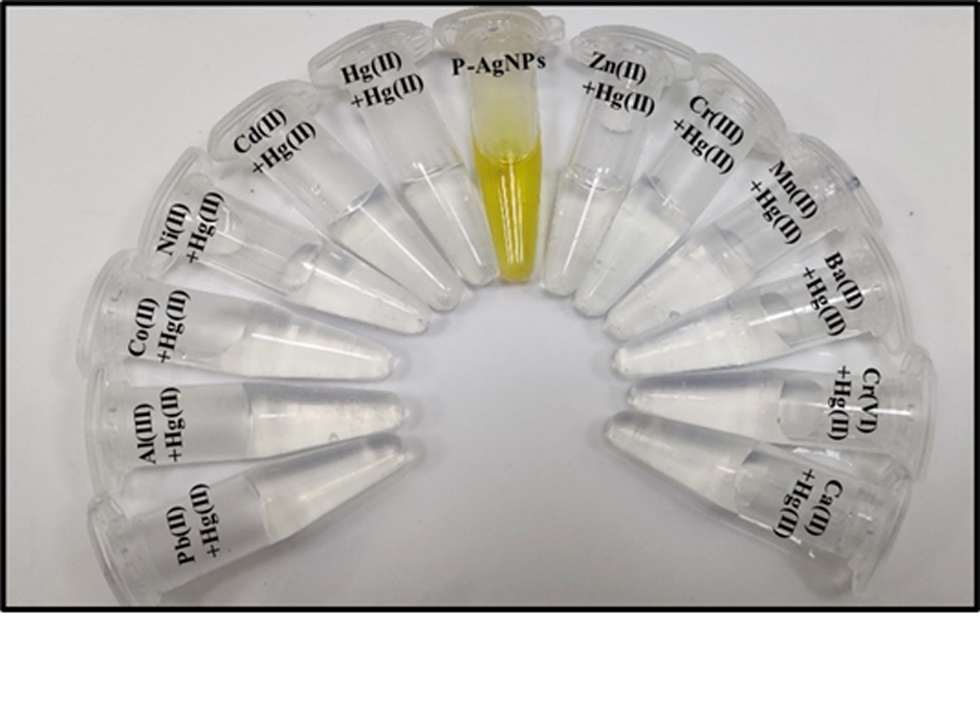


**Fig S3.** Interference study of P-AgNPs solution containing Hg^2+^and other metal ions
